# Resource Utilisation and Costs of Depressive Patients in Germany: Results from the Primary Care Monitoring for Depressive Patients Trial

**DOI:** 10.1155/2014/730891

**Published:** 2014-09-09

**Authors:** Christian Krauth, Jona T. Stahmeyer, Juliana J. Petersen, Antje Freytag, Ferdinand M. Gerlach, Jochen Gensichen

**Affiliations:** ^1^Institute for Epidemiology, Social Medicine and Health System Research, Hannover Medical School, Carl-Neuberg-Street 1, 30625 Hannover, Germany; ^2^Institute for General Practice, Goethe-University Frankfurt, Theodor-Stern-Kai 7, 60590 Frankfurt am Main, Germany; ^3^Institute of General Practice and Family Medicine, Friedrich-Schiller-University Jena, Bachstraße 18, 07743 Jena, Germany

## Abstract

*Background.* Depression is the most common type of mental disorder in Germany. It is associated with a high level of suffering for individuals and imposes a significant burden on society. The aim of this study was to estimate the depression related costs in Germany taking a societal perspective. *Materials and Methods.* Data were collected from the primary care monitoring for depressive patients trial (PRoMPT) of patients with major depressive disorder who were treated in a primary care setting. Resource utilisation and days of sick leave were observed and analysed over a 1-year period. *Results.* Average depression related costs of €3813 were calculated. Significant differences in total costs due to sex were demonstrated. Male patients had considerable higher total costs than female patients, whereas single cost categories did not differ significantly. Further, differences in costs according to severity of disease and age were observed. The economic burden to society was estimated at €15.6 billion per year. *Conclusion.* The study results show that depression poses a significant economic burden to society. There is a high potential for prevention, treatment, and patient management innovations to identify and treat patients at an early stage.

## 1. Background

Depression is the most common type of mental disorder among adults in western countries [1]. The German Health Interview and Examination Survey for adults (DEGS1-MH) determined a 12-month prevalence of 7.7% for unipolar depression, 6.0% for major depression, and 2.0% for dysthymia in Germany [2, 3]. The lifetime prevalence of any depressive disorder is considerably higher (17.1%) [4, 5]. According to the Global Burden of Disease study, mental and substance use disorders were fifth leading disorder category of global disability-adjusted life years (DALYs). Further, mental and substance use disorders were the leading cause of all nonfatal burden of disease expressed in terms of years lived in disability (YLDs). In both outcomes, depressive disorders amount for the largest part of disease burden [6]. Almost like no other illness, depression is associated with a high level of suffering and reduction in quality of life and imposes a huge burden on individuals, family members, and economies as well. A recent analysis has shown considerable deficits in diagnosing and treating depressive disorders in Germany. A large part of depressive disorders is not diagnosed as routine screening or standardised diagnostic instruments are rarely used. The minority of patients receives an adequate guideline based treatment [7–9]. Depressive patients utilise health care services more frequently and produce higher costs than people without depression [10–12]. Several international studies have estimated the burden of depression in various contexts and have shown the costs arising from this disorder. A systematic review by Luppa et al. provides a good overview on cost of illness studies [13]. In Germany, different studies have estimated the costs of depressive disorders with different data sources in various contexts [11, 12, 14]. However, information on indirect cost (productivity losses) is not available yet. As depression is associated with restrictions in social functioning productivity losses are of major importance estimating the cost of illness. The relevance of depression related productivity losses is shown in several international studies. Indirect costs account for over 60% of the economic burden of depression in the USA [15]. Similar conclusions were drawn by Ekman et al. and Sobocki et al. who estimated the costs of depression in Sweden. Productivity losses due to sick leave and early retirement accounted for 88% and 86% of total depression related costs, respectively [16, 17]. A British study by Thomas and Morris underlined the relevance of indirect costs. This study estimated the indirect cost to be over 90% of total costs [18].

Mental disorders are the leading cause for early retirement in Germany. About one third of all early retirements in 2007 are attributed to mental and behavioural disorders. Further, major depression is a leading cause for sick leave and is associated with long work disability periods. In 2012, the average work disability period due to a single depressive episode (ICD-10, F32) was 46.4 days and 64.6 days for a recurrent depressive episode (ICD-10, F33) [19, 20].

The objective of the study is to analyse the resource utilisation and costs of depressive patients in Germany. Subgroup analyses were conducted for sex, age, and severity disease. In addition, a projection of the burden to society is stated.

## 2. Materials and Methods

### 2.1. Cost of Illness Studies

The aim of cost of illness studies is to identify and measure all costs caused by a specific disease [21]. Cost of illness studies is performed to illustrate the burden of a disease on society in monetary terms [22]. Policymakers use this type of study for justifying budgets, setting priorities in funding research, or developing intervention programmes to ameliorate and prevent diseases [23]. Cost of illness studies can give precise information in which areas of healthcare provision costs incur and where saving potentials may occur. Furthermore, cost of illness studies enables focusing on specific indications and patient groups. Different subgroups can be analysed in order to detect differences with respect to sociodemographic or clinical characteristics.

Cost of illness studies can be performed using different methodical approaches. The perspective chosen for the evaluation is of particular importance and needs to be adjusted for study objectives. Analyses can be carried out adopting the patients', payers', health care system, or societal perspective [24]. Cost of illness studies can be performed using a prevalence or incidence based approach. The prevalence approach is the most common methodology and estimates the total burden of a disease in a given period of time. The incidence based approach calculates the lifetime costs resulting from a disease [21, 23].

Resource utilisation and costs can be measured using a top-down or bottom-up methodology. In the top-down approach aggregated data from national registries and official statistics are used to quantify the expenditures or burden of a disease. This approach uses highly aggregated data such as claims data. In most cases information is only available for aggregated disease groups. In the bottom-up approach, data are collected directly from a sample of patients or patient records. With this approach, specific types of illnesses and subgroups (e.g., depending on disease stage) can be analysed more easily. For the estimation of the societal burden, data has to be extrapolated using national prevalence data.

### 2.2. Data Source

For the present study a prevalence-based bottom-up approach was used to identify depression related disease specific resource utilisation and costs. Costs were determined taking a societal perspective. Data were obtained from the PRoMPT (primary care monitoring for depressive patient's trial) project. This cluster randomized controlled trial examined whether case management provided by practice based health care assistants in general practitioner (GP) practices is effective in reducing the symptoms and adherence of patients with depression. The intervention group received case management over one year and the control patient group received usual care. The primary outcome parameter was depression symptoms. Secondary outcome parameters were adherence, quality of life, health care utilisation, perceived quality of care, and days out of work. Study design was approved by the ethics commission of the University of Frankfurt [25, 26].

A total of 70 GPs located in the federal state of Hesse participated in the study. Patients already on treatment were screened by GPs in consecutive order of appearance in the practice and in case of depression informed about the option to participate in the study. Inclusion criteria for patients were diagnosis of major depression (PHQ-D screening confirmed in clinical interview) with indication for antidepressive treatment, aged from 18 to 80 years, ability to give informed consent, and sufficient knowledge of the German language. Patients who were suicidal or addicted to alcohol were excluded. In total, 626 patients were included in the study, 99 patients dropped out [26]. Data was collected from patients at three times: baseline (T0), follow-up after 6 months (T1) and follow-up after 12 months (T2). Sociodemographic and clinical data were assessed by means of questionnaires. Data for resource utilisation including general practitioner, medical specialist contacts, psychotherapy, hospitalisation, and prescribed medication was collected using patient questionnaires. Additional utilisation data was collected from the patients' medical records. Detailed information on study design has been published elsewhere [25, 26].

Complete data (T0, T1, and T2) was not available for all patients. Further, time points of cost measurement deviated considerably from planned intervals. In order to cover resource utilisation of a one year period appropriately, patients were included in the analysis if an interval of five to seven months between time points of measurement was observed. If intervals of measurement were shorter than five months or longer than seven months, patients were excluded as a full one year period could not be observed appropriately. In total, data of 263 patients were analysed in this study.

### 2.3. Cost Analysis

The analysis is based on direct and indirect costs due to depressive disorder. Direct costs are based on healthcare utilisation and were observed over a 1-year period. This included

- Medication,
- appointments with general practitioner,
- appointments with medical specialists,
- psychotherapy,
- hospitalisation.

The calculation of indirect cost is based on days of sick leave due to depressive disorder. Sick leave was certified by general practitioner or medical specialist. Information on early retirement was asked but could not clearly be attributed to the depressive disorder. Indirect costs only represent costs due to sick leave.

For the valuation of health care utilisation recommendations of AG MEG (Working Group Methods in Health Economic Evaluation) are used [27]. The monetary valuation of antidepressive medication was performed using pharmacy prices. Prices were adapted from the German drug directory “Lauer-Taxe” and adjusted for pharmacy and producer discount for statutory health insurance members. Missing doses (<1%) were replaced by defined daily dose (DDD) [28].

The cost of outpatient care was calculated using average cost for doctor visits. Data from AG MEG were extrapolated to the year 2006 [27]. A general practitioner visit was valued at €16.06. Appointments with medical specialists are valued at €34.86/visit. As no specifications for psychotherapy were made in the survey, average costs for a psychotherapy session were used. According to information given by the German Society for Behaviour Therapy and the National Chamber of Psychotherapists psychotherapy sessions are valued with average costs of €70.

Cost for hospital stays were calculated, using data from the Federal Statistical Office. The cost of an average day in hospital amount €408 in 2006 without investment costs and cost of capital [29]. Total cost of €464.11 per hospital day result, adding investment cost and cost of capital of €56.11 per day [27].

Indirect costs were estimated using the human capital approach [30]. The valuation of productivity losses is based on labour costs. Data from the Federal Statistical Office were analysed and average labour costs of €90.77 per calendar day were calculated applying average labour costs per month in 2006 [29]. Indirect costs were only calculated for the working population.

### 2.4. Subgroups

In order to estimate differences in the study population according to sociodemographic and clinical criteria, subgroup analyses are performed. Participants are classified by sex, age, and severity of depression. For the distinction according to age, four age groups were generated: 18–35 years, 36–50 years, 51–64 years, and ≥65 years. Severity of depression was assessed using the Patient Health Questionnaire. The PHQ-9 is a nine-item depression scale and validated in primary care use. It is based on diagnostic criteria for major depressive disorder in the DSM-IV. Each item is scored from 0 (not at all) to 3 (nearly every day). The score ranges from 0 to 27 and higher scores indicate more severe depression. Severity is divided in five different categories: 0–4 no depression, 5–10 minimal symptoms for major depression, 10–14 mild major depression, 15–19 moderately severe major depression, and ≥20 severe major depression [31].

### 2.5. Statistical Analyses

For statistical analysis IBM SPSS Statistics 22 was used. Quantitative values are indicated in mean and statistical differences were assessed by using Student's *t*-test or ANOVA. For qualitative data Chi-square-test was used. Due to skewed distributions of costs bootstrapped data was used for pairwise comparison of differences in mean costs. Differences were considered significant at a level of *P* ≤ 0.05.

## 3. Results

### 3.1. Study Population and Resource Utilisation

Sociodemographic and clinical criteria as well as resource utilisation were evaluated for 263 participants. 199 patients were female (75.7%), and 64 were male (24.3%). The average age was 52 years (SD 13.9). Type of health insurance was known for 84.8% of the population. Of these, 97.3% were legally health insured and 2.7% privately insured. An average PHQ-9 score of 17.2 (SD 3.5) was measured at baseline. Severe major depression was diagnosed with 66 patients (25.1%). The main part of the study population suffered from moderately severe major depression (*n* = 136, 51.7%). Mild major depression was diagnosed in 60 patients (22.8%), one patient suffered from minimal depressive symptoms (0.4%). At baseline, 49.8% of participants were employed. 24 participants (9.1%) received pension based on reduction in earning capacity. Other patients were unemployed (11.8%), housewife/house husband (8.8%) or pensioners (19.5%). Most patients diagnosed with major depression suffered from one or more diseases. The most common illnesses were endocrine, nutritional, and metabolic diseases and diseases of the circulatory system.

General practitioners were consulted 17.6 (SD 14.3) times a year in average. Medical specialists were consulted 3.2 (SD 5.5) times a year. Patients had an average number of psychotherapy sessions of 10.2 (SD 19.6). Regarding medication, antidepressants (76%) were prescribed most common by doctors, followed by psycholeptics with 14% of total prescriptions. Other medications only represented a small portion for antidepressive treatment prescriptions (10%). More than two-thirds of treated patients received antidepressive medication (68.8%). Because of their mental illness 26 patients (9.9%) were treated as inpatients. In average, these patients were treated 31.4 (SD 23.7) days in hospital during the 1-year period.

### 3.2. Total Cost

Total depression related cost were €3813 (SD 7851) per patient. Direct costs sum up to €2750 (72% of total costs, SD 5852) and indirect costs to €1063 (28% of total costs, SD 4067). Among direct costs, inpatient care accounted for the largest part (€1438; SD 5523). Expenditure in psychotherapy amounts for €714 (SD 1369). The cost of outpatient care (general practitioner and medical specialists) was estimated as €393 (SD 316). Drug costs due to depressive disorder were estimated as €204 (SD 308) per year. Antidepressants account for the largest part of drug costs. Costs are shown in Table 1.

### 3.3. Subgroup Analysis

The analysis by sex showed significant differences in total costs (*P* = 0.032). On average, male patients caused more than double the cost of female patients (male: €6308; female: €3010). A breakdown analysis showed no significant differences for single cost categories (pharmaceuticals, outpatient care, psychotherapy, inpatient care, and sick leave). Detailed results are presented in Table 1.

**Figure 1:**
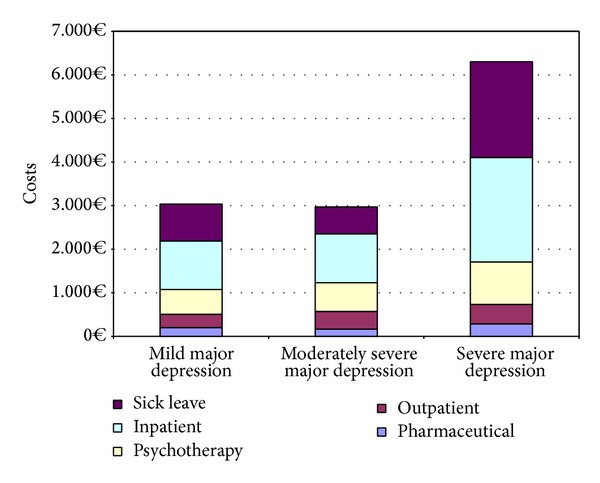
Total disease-related cost per patient by severity.

In order to explore connections between severity of depression and costs, a subgroup analysis by different severity codes is performed. One patient with minimal depressive symptoms was excluded, so that 262 patients were analysed in three groups. The analysis showed significant differences in total costs due to severity of depression (*P* = 0.011). The pairwise comparison of mean difference confidence intervals for bootstrapped data showed significantly higher costs in patients with severe major depression (€6302) compared to patients with moderately severe major depression (€2971). The analysis of the different cost areas showed significant differences in pharmaceutical costs between patients with severe major depression and patients with moderately severe major depression. Ambulatory care costs were significantly lower in patients with mild major depression compared to severe stages of disease. Sick leave costs were significantly higher in patients with severe major depression compared to patients with moderately severe major depression. Costs are shown in Figure 1.

Total costs were significantly different between the four age groups (*P* = 0.021). The pairwise comparison of mean difference confidence intervals for bootstrapped data showed significant lower mean total costs in the oldest age group (65–85 years) compared to age groups 36–50 years and 51–64 years. The huge cost differences of the oldest group compared to the younger age groups are partly caused by sick leave costs which are not charged in this age group. A breakdown analysis showed significant differences in pharmaceutical costs between age groups 18–35 years and 51–64 years. Outpatient care costs were significantly higher and psychotherapy costs lower in patients aged 65–85 years compared to all other age groups.

The costs of the different age groups are shown in Table 1.

### 3.4. Costs of Major Depression in Germany

**Figure 2:**
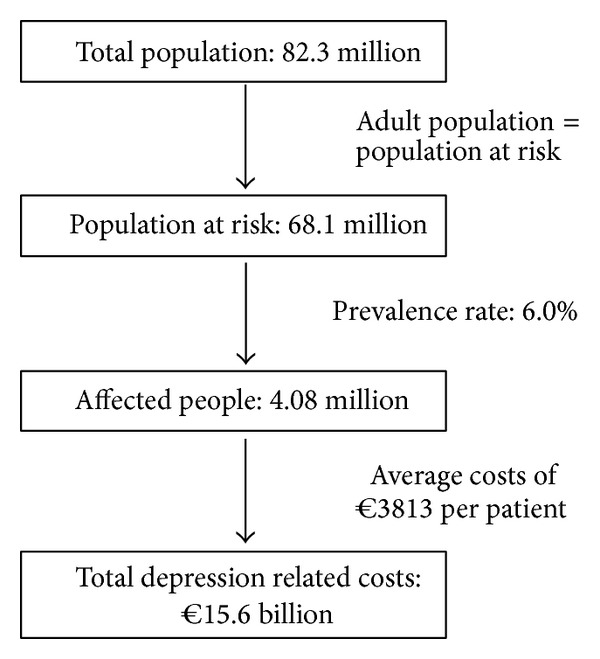
Estimated total costs of depression in Germany.

The calculation of the burden caused by depressive disorders in Germany in 2006 is based on the results of average depression related costs per patient (€3813) and prevalence rates estimated in the German Health Interview and Examination Survey (GHS). Population at risk consists mainly of adult population. Assuming a prevalence rate of 6.0% [2, 3] and population at risk of 68 million [29], a number of 4.08 million affected people are estimated. From these data total depression related costs of €15.6 billion in Germany are calculated (Figure 2).

## 4. Discussion

This study used a bottom-up approach to investigate the cost of patients suffering from major depression in a primary care setting over a 1-year period. It is one of few studies where the impact of disease severity on costs is analysed. Additionally, differences according to sex and age were shown. The analyses included 263 patients. No differences with respect to sex, age, and PHQ-9 score in comparison to total population were observed.

This is the first study which attempts to calculate productivity losses due to depression in Germany. Unfortunately, occupational disability and early retirement could not clearly be attributed to the depressive disorder, so that indirect costs only represent morbidity costs due to sick leave. Mortality costs were also not considered. This probably leads to an underestimation of indirect costs, as indirect costs represent a huge share of total costs in international studies [15, 18, 32]. If early retirement could clearly be attributed to the depressive disorder, additional morbidity costs of €2847 have to be added. Including indirect costs of early retirement, average total costs would increase to €6660 per patient. Direct costs share a part of 41% and indirect costs of 59%. Rehabilitative services were meant to be documented in the context of a psychiatric inpatient setting. Nevertheless, healthcare service utilisation questionnaire did not explicitly ask for rehabilitative services such as ergotherapy and physiotherapy in ambulatory care, which may lead to a underestimation of direct costs.

In order to analyse how uncertainty affects the results, cost parameters were analysed using the 95% confidence interval. Beside the base case, the lower limit and the upper limit can represent a best-case and a worst-case scenario (Table 2).

Male patients showed considerably higher costs than female patients, whereas single costs categories did not significantly differ. Costs are mainly influenced by few patients utilising a huge amount of health services. Costs of psychotherapy and inpatient care were about twice as high. These figures correspond with gender differences reported by public statistics of mental health: the lifetime prevalence for depression for adults was 15.4% in women and only half in men (7.8%) [33]. At the same time, men in 2012 accounted for almost the same amount of inpatient stays due to psychic illnesses (ICD-10 codes F00-F99) per 100000 inhabitants than women (1704 versus 1679) [34]. This might be interpreted as men tend to show a lower awareness for health issues, tend to suppress depression symptoms, tend to postpone treatment, and to develop to more serious cases, finally needing more intensive therapy like inpatient care. The excess of indirect costs due to depression in men over women might be explained by the generally higher full-time employment rate in men than in women in Germany which was also observed in our study [35]. Patients from 65 to 80 years showed considerably lower costs for psychotherapy. A corresponding observation is the lower cost of inpatient care due to depression in elder patients. The fact that elder people still make low use of psychotherapy has been described for Germany [36]. A major impeding factor seems to be the tendency in the elderly to underreport depression symptoms [37].

The underlying resource utilisation data is derived from the ProMPT trial. Previous analyses may differ because different cost-estimates were used [38]. Due to study objective, patients were initially separated into two groups, intervention group and control group. Within the cost analysis all patients were analysed, regardless of previous group. The comparison of the treatment groups allows the identification of differences in costs caused by treatment. Differences were not significant. A breakdown analysis also showed no significant differences in the cost areas. Additional cost for implementing and performing case management treatment were not included in the analysis. Average intervention costs were €276 [38]. Although no differences in resource utilisation and costs were observed, case management treatment had a positive effect on the course of the disease. Intervention recipients had significantly lower PHQ-9 scores, higher depression treatment response rates, and an increased adherence to antidepressant medication than control patients [26].

This study estimates yearly costs of €3813 per affected patient (direct costs: €2750; indirect costs: €1063). Previously published studies by Salize et al. and Friemel et al. estimated direct costs of €2073 and €686 per patient in Germany [39, 40]. A comparison of the direct cost is complicated as considerable methodical differences exist. Whereas only patients with major depression according to DSM-IV criteria were included in the present study, Friemel et al. also included patients with minor depression and dysthymia. Further, not all patients have used medical services. Considering only patients who received medical services, direct costs of €1264 were estimated by Friemel et al. Study population in the study of Salize et al. consists of patients who were diagnosed with a depressive disorder by the treating physician. By the application of ICD-10 criteria for depressive disorder, average direct costs increase to €2541, which comes close to the estimated direct costs of the present study. Besides varieties in study population, differences in resource valuation and study period are found. Salize et al. developed a unit cost approach for average costs per patient visit. Contact rates and average costs per visit were not stated. Resource utilisation was observed over an 8-week period and extrapolated on yearly basis. Friemel et al. calculated costs using a cost per minute approach. In older patients a recent claims data analysis showed average annual depression related direct costs of €487 [14]. Another study showed an increased resource utilisation and costs (depressed: €8,145 versus nondepressed: €3,137) in older patients with depression [11].

A study by Sobocki et al. used a top-down approach to estimate the cost of depression in Sweden. Total costs of depression in Sweden at €3.5 billion in 2005 were calculated. Indirect costs represent about 86% of total costs [32]. A more recent study confirmed these results showing annual costs of €17,279, 88% of these indirect costs [17]. The cost of depression among adults in England in 2000 was calculated by Thomas and Morris. They estimated yearly costs of over £9 billion. Indirect costs represent 96% of the total costs [18]. A further study by Greenberg et al. estimated a total economic burden of illness $83.1 billion in 2000. Of this, direct treatment costs of $26.1 billion ($3309 per case) were estimated. Indirect costs including suicide related costs and workplace costs sum up for $56.9 billion in total or $4809 per case.

Although there can be found many studies on cost of depression in the international literature, comparability is limited. A systematic review by Luppa et al. showed wide methodical differences in cost of illness studies of depression. Main differences can be found in study approach (top-down versus bottom-up), cost approach (depression-related costs versus total health care costs), and identification of depressed cases [13]. Further, study results are affected by various factors such as study country, health care system, study design, or time. As there is no standard procedure for identification, measurement, and valuation of resources, comparability is made even more difficult [41, 42].

## 5. Conclusion

The cost of depression poses a significant burden to society and is associated with a high level of suffering for individuals. Severe courses result in significant higher costs. Taking this into account, there is need to recognise and treat patients with depression at an early stage. There is high potential for improving prevention, patient detection, and treatment. Newly introduced concepts must pursue the objective to improve healthcare provision for depressive patients and thereby reduce costs.

## Figures and Tables

**Table 1:** Costs of different subgroups (in Euro); number in brackets shows bootstrapped 95% confidence intervals.

Base Case		Sex		Age (years)				Severity		
		Male	Female	18–35	36–50	51–64	65–85	Mild major depression	Moderately severe major depression	Severe major depression
**Type of costs**	*n* = 263	*n* = 64	*n* = 199	*n* = 27	*n* = 91	*n* = 95	*n* = 50	*n* = 60	*n* = 136	*n* = 66

Pharmaceutical	204 (171–242)	213 (145–295)	202 (163–248)	126 (62–205)	198 (137–263)	234 (170–310)	202 (133–291)	201 (134–279)	167 (128–211)	286 (197–385)
Outpatient	393 (358–433)	380 (307–458)	397 (354–439)	330 (245–428)	354 (297–426)	386 (327–461)	511 (429–609)	305 (240–376)	405 (353–462)	447 (368–533)
Psychotherapy	714 (565–886)	1073 (614–1588)	599 (462–755)	879 (451–1384)	855 (600–1130)	826 (527–1200)	158 (38–312)	568 (324–833)	658 (475–871)	975 (525–1541)
Inpatient	1438 (833–2199)	2415 (895–4334)	1124 (515–1826)	2355 (13–5776)	2239 (972–3921)	806 (149–1663)	687 (119–1550)	1114 (82–2684)	1126 (507–1894)	2398 (690–4519)

**Direct costs**	**2750**	**4081**	**2322**	**3690**	**3646**	**2252**	**1559**	**2189**	**2355**	**4106**

Sick leave costs	1063 (637–1634)	2227 (795–3860)	688 (363–1116)	491 (78–1121)	1979 (932–3416)	906 (331–1609)	0 (0-0)	847 (82–2178)	615 (203–1319)	2196 (1072–3587)

**Indirect costs**	**1063 **	**2227 **	**688**	**491**	**1979**	**906**	**0**	**847**	**615**	**2196 **

**Total costs**	**3813 (2963–4967)**	**6308 (3681–9374)**	**3010 (2223–3880)**	**4181 (1416–7942)**	**5625 (3721–8175)**	**3159 (2152–4364)**	**1559 (856–2536)**	**3036 (1413–5236)**	**2971 (2117–4105)**	**6302 (3914–9242)**

**Table 2:** Cost scenarios (in Euro).

Type of costs	Base case	Best case	Worst case
Pharmaceutical	204	167	242
Outpatient	393	355	431
Psychotherapy	714	548	881
Inpatient	1438	768	2109

**Direct costs**	**2750 **	**1837 **	**3663 **

Sick leave costs	1063	569	1556
Occupational disability	2847	1728	3965

**Indirect costs**	**3910 **	**2297 **	**5521 **

**Total costs**	** 6660 **	**4134 **	**9184 **
